# Diabetic Endothelial Cells Differentiated From Patient iPSCs Show Dysregulated Glycine Homeostasis and Senescence Associated Phenotypes

**DOI:** 10.3389/fcell.2021.667252

**Published:** 2021-05-31

**Authors:** Liping Su, Xiaocen Kong, Sze Jie Loo, Yu Gao, Jean-Paul Kovalik, Xiaofei Su, Jianhua Ma, Lei Ye

**Affiliations:** ^1^National Heart Centre Singapore, National Heart Research Institute Singapore, Singapore, Singapore; ^2^Department of Endocrinology, Nanjing First Hospital, Nanjing Medical University, Nanjing, China; ^3^Department of Cardiology, Ren Ji Hospital, School of Medicine, Shanghai Jiao Tong University, Shanghai, China; ^4^Programme in Cardiovascular & Metabolic Disorders, Duke-National University of Singapore, Singapore, Singapore

**Keywords:** endothelium, endothelial function, senescence, mitochondrial function, glycine

## Abstract

Induced pluripotent stem cells derived cells (iPSCs) not only can be used for personalized cell transfer therapy, but also can be used for modeling diseases for drug screening and discovery *in vitro.* Although prior studies have characterized the function of rodent iPSCs derived endothelial cells (ECs) in diabetes or metabolic syndrome, feature phenotypes are largely unknown in hiPSC-ECs from patients with diabetes. Here, we used hiPSC lines from patients with type 2 diabetes mellitus (T2DM) and differentiated them into ECs (dia-hiPSC-ECs). We found that dia-hiPSC-ECs had disrupted glycine homeostasis, increased senescence, and impaired mitochondrial function and angiogenic potential as compared with healthy hiPSC-ECs. These signature phenotypes will be helpful to establish dia-hiPSC-ECs as models of endothelial dysfunction for understanding molecular mechanisms of disease and for identifying and testing new targets for the treatment of endothelial dysfunction in diabetes.

## Introduction

Vascular endothelial cells (ECs) play an important role in maintaining vascular homeostasis (Rajendran et al., 2013). In addition to serving as a physical barrier between the vessel wall and blood, ECs regulate platelet aggregation, coagulation, fibrinolysis, and vascular tone through secreting nitric oxide (NO), cytokines, and growth factors (Kruger-Genge et al., 2019). Endothelial dysfunction is a condition in which the EC loses its physiological properties, promotes vasoconstriction, fibrinolysis, inflammation, and atherogenesis (Kruger-Genge et al., 2019).

Type 2 diabetes mellitus (T2DM) is characterized by hyperglycemia, insulin resistance, and relative lack of insulin secretion (DeFronzo et al., 2015). T2DM causes endothelial dysfunction, induces abnormal aniogenesis, and changes serum amino acid metabolism, especially for glycine, which is significantly decreased, due to hyperglycemia (Thalacker-Mercer et al., 2014; Drabkova et al., 2015; Giraldo-Grueso and Echeverri, 2020). These factors constitute molecule and cellular basis for both microvascular and macrovascular complications in diabetes (DeFronzo et al., 2015; Palmer A.K. et al., 2015; Guo et al., 2017; Yan-Do and MacDonald, 2017; Alves et al., 2019; Rashvand et al., 2019; Giraldo-Grueso and Echeverri, 2020). Treatments to improve endothelial function, cell viability, and angiogenesis and correct glycine metabolism may be promising therapeutic targets for preventing microvascular and macrovascular complications in diabetes.

Although primary human ECs are the best choice of cells for studying endothelial dysfunction, senescence, and angiogenesis, they are limited by cell source and cell isolation technique. The discovery of human induced pluripotent stem cells (hiPSCs) has paved the way for EC generation (Patsch et al., 2015; Zhang et al., 2017; Christensen et al., 2019). Disease specific hiPSC lines can be reprogrammed from patient somatic cells and subsequently can be differentiated back into somatic cells for disease modeling and drug screening and discovering *in vitro* (Liu et al., 2018; Rowe and Daley, 2019; Williams and Wu, 2019).

In this study, we investigated hiPSC lines reprogrammed from dermal fibroblasts derived from patients with T2DM. ECs were generated from diabetic hiPSCs (dia-hiPSCs). We aim to identify signature and specific phenotypes in dia-hiPSC derived ECs (dia-hiPSC-ECs). This will be helpful to establish dia-hiPSC-ECs as models for understanding the molecular mechanism as well as screening and discovering drugs for treatment of endothelial dysfunction and senescence, abnormal angiogenesis, and disrupted glycine homeostasis in diabetes.

## Materials and Methods

### hiPSC Culture and Differentiation

Four hiPSC lines used in this study were reprogrammed from dermal fibroblasts using Sendai virus and 4 reprogramming factors (Thermo Fisher, North Logan, UT, United States): OCT4, SOX2, KLF4, and C-MYC, as described previously (Ye et al., 2013). PCBC16iPS and P1C1iPS cells were reprogrammed from human neonatal and adult dermal fibroblasts of healthy subjects (Lonza, Switzerland), respectively (Zhang et al., 2014). DP2-C8iPS and DP3-C6iPS cells were reprogrammed from dermal fibroblasts of two adult patients with T2DM. PCBC16iPS and P1C1iPS are derived from male donors. DP2 donor was a female patient and 53 years old, while DP3 donor was a male patient and 51 years old. The procedures were approved by the ethics committee of Nanjing Hospital, Nanjing, China and the Centralised Institutional Review Board of Singapore Health Services Pte. Ltd, Singapore. Informed consent forms were signed by both patients. hiPSCs were cultured in a feeder-free system with a 1:1 mixture of E8/mTeSR (STEMCELL Technologies, Canada) and passaged every 4 days with Versene (Thermo Fisher Scientific, United States). PCBC16iPS was an established hiPSC line and described previously (Ye et al., 2013, 2014; Zhang et al., 2014; Su et al., 2018; Tan et al., 2019; Tao et al., 2020).

Pluripotency of P1C1iPSC, DP2-C8iPSC, and DP3-C6iPSC were characterized by fluorescence immunostaining, teratoma formation assay, and karyotyping. For fluorescence immunostaining, hiPSCs were fixed with 4% paraformaldehyde for 20 min at room temperature followed by 0.1% triton X-100 permeabilization. After blocking, primary antibody, mouse anti-Oct3/4 (SC-365509, Santa Cruz Biotech, United States) or anti-TRA-1-60 (130-106-872, Miltenyi Biotec, Germany), at 1:100 dilution each was added into the DPBS containing 10% goat serum (Thermo Fisher Scientific, United States) (10% DPBS) and incubated overnight at 4°C. Then, cells were incubated with FITC-conjugated donkey anti-mouse IgG secondary antibody (Thermo Fisher Scientific, United States) in 10% DPBS for 1 h at room temperature, labeled with 4′,6-diamidino-2-phenylindole (DAPI, Sigma-Aldrich, United States), washed, and viewed under a fluorescence microscope (Olympus, Japan).

For teratoma formation experiment, the animal experimental protocol and procedures were approved by the Institutional Animal Care and Use Committee of Singapore Health Services Pte. Ltd. 2 × 10^6^ hiPSCs were injected into the flanks of NOD-SCID mice (Invivos, Singapore). Animals were followed-up to 3 months. Teratoma were explanted, processed, and embedded into paraffin. Tissue sections of teratoma were stained with hematoxylin and eosin and viewed under an Olympus microscope to identify endoderm, mesoderm, and ectoderm cells. For karyotyping, hiPSCs were assessed by Cytogenetics Lab. of Department of Molecular Pathology, Singapore General Hospital, Singapore or of KK Women’s and Children’s Hospital, Singapore.

The EC differentiation protocol was described previously by us with modification (Zhang et al., 2014; Su et al., 2018). Briefly, 2-3 days before initiating differentiation, hiPSCs were dissociated into single cells and seeded into 6-well plates. On day 0 of differentiation, cells were cultured in DMEM/F12 medium (Thermo Fisher Scientific, United States) supplemented with B27 without insulin (B27-, Thermo Fisher Scientific, United States) and 10 μM CHIR99021 (CHIR, STEMCELL Technologies, Canada) and 1 μM U46619 (Santa Cruz Biotech, United States) for 24 h. On day 1, medium was replaced with DMEM/F12 supplemented with B27-, 100 ng/ml vascular endothelial growth factor-165 (VEGF) (ABM, Canada), 10 ng/ml transforming growth factor β1 (TGFβ1, ProSpec-Tany Tech., Israel), and 50 ng/ml erythropoietin (EPO, ProSpec-Tany Tech., Israel) for 48 h, the medium was refreshed on day 3, and the cells were cultured for another 48 h. On day 5, the differentiating hiPSCs were harvested and cultured in EGM2-MV (Lonza, Switzerland) supplemented with B27, 100 ng/ml VEGF, and 10 μM SB-431542 (SB, MedChemExpress, United States) in flasks that were coated with 1 μg/cm^2^ fibronectin (Sigma-Aldrich, United States). The medium was changed every 2 days. ECs were purified on day 11 using fluorescence activated cell sorting (FACS). Cells positive for both CD31 and CD144 (both from BD Pharmingen, United States) expression were collected and expanded (Zhang et al., 2014; Su et al., 2018). ECs were cultured in endothelial growth medium (EGM) which was composed of EGM2-MV medium supplemented with B27, 100 ng/ml VEGF, and 10 μM SB (Zhang et al., 2014).

### Characterization of hiPSC-ECs *in vitro*

#### Fluorescence Immunostaining

For characterization of hiPSC-ECs *in vitro*, cells were fixed with 4% paraformaldehyde for 20 min at room temperature. After blocking, primary antibodies, mouse anti-CD31 or anti-CD144 (both from BD Pharmingen, United States), at 1:100 dilution was added into 10% DPBS and incubated overnight at 4°C. Then, cells were incubated with PE-conjugated goat anti-mouse IgG secondary antibody (Thermo Fisher Scientific, United States) in 10% DPBS for 1 h at room temperature, labeled with DAPI, washed, and viewed under a fluorescence microscope (Olympus, Japan).

#### Cell Population Doubling Time

Endothelial cell population doubling time was calculated within 7 days post-sorting. Briefly, ECs were harvested on day 2 post-sorting and cultured in 6-well plates (2×10^5^ cells/well) in EGM. The medium was changed every 2 days. ECs were harvested and counted on day 7.

#### Western Blot Analysis

Endothelial cells on day-7 post-sorting were used for Western Blot analysis as described previously (Ye et al., 2009; Su et al., 2018). For protein analysis using EC supernatant, 2 × 10^5^ ECs/well were cultured in 1 ml EGM for 48 h. Then supernatant was collected to determine the endothelin-1 and Matrix metalloproteinase-1 (MMP-1) proteins secreted from ECs. Cell lysate was prepared using PhosphoSafe^TM^ Extraction Reagent (Merck, Germany) and protein concentrations were determined using Bradford reagent (Bio-Rad Laboratories, United States). Proteins from supernatant or cell lysate were separated, electrophoretically blotted onto nitrocellulose membranes, and washed with 10 mM Tris-HCl buffer (pH 7.6) containing 0.05% Tween-20; then, the membranes were incubated in blocking buffer (5% non-fat dry milk, 10 mM Tris pH 7.5, 100 mM NaCl, 0.1% Tween-20) at room temperature for 1 h and with diluted primary antibodies for overnight at 4°C. Primary antibodies were detected with secondary antibodies conjugated with horseradish peroxidase (HRP) and visualized with a ChemiDoc^TM^ MP Imaging System (Bio-Lab, United States) and Image Lab 5.1 software (Bio-Lab, United States). The detailed antibody information was provided in Supplementary Table 1.

### Endothelial Function *in vitro*

#### Dil-Conjugated Acetylated Low-Density Lipoprotein (Dil-ace-LDL) Uptake and Tube Formation

Dil-ace-LDL uptake and tube formation were evaluated as described previously (Zhang et al., 2014; Su et al., 2018). For DiI-ace-LDL uptake assay, hiPSC-ECs were incubated with DAPI overnight (1:1,000 dilution) and then in EGM supplemented with 10 μg/mL of DiI-ace-LDL (Life Technologies, United States) at 37 °C for 12 h. For tube-formation assay, cells were seeded in 48-well plate that had been coated with Matrigel (Corning, United States) and incubated at 37°C for 24 h. Numbers of node, junction, and branches and total branches length per magnification (10×) were quantified using angiogenesis analyzer of Image J.

#### Nitric Oxide (NO) Produced by ECs

To assess NO produced, hiPSC-ECs on day 7 post-sorting were seeded in 12-well plate and cultured in 1 ml EGM for 48 h. Supernatant was collected and stored at –80°C. Cells were washed with DPBS and lysed in 100 μl PhosphoSafe^TM^ Extraction Reagent. The concentration of NO was determined using Nitric Oxide Detection Kit as per instruction (SKT-212, StressMarq Biosciences Inc., United States) and normalized by protein quantity and expressed as μM/mg.

#### Endothelin-1 Secreted by ECs

To determine secreted endothelin-1, a vasoconstrictor, hiPSC-ECs supernatant was collected for Western Blot analysis as described above. Endothelin-1 protein expression was presented as fold change after comparing with that of PCBC-EC which was considered as 100%. The detailed information on endothelain-1 antibody was provided in Supplementary Table 1.

#### Adhesion Molecule Expressed by ECs

Protein expression of adhesion molecule, including ICAM-1 and VCAM-1, was determined using Western Blot as described above. The protein expression level was normalized by GAPDH and expressed as percentage of GAPDH. The detailed information on ICAM-1, VCAM-1, and GAPDH antibodies was provided in Supplementary Table 1.

### Senescence of ECs

To determine cell senescence, ECs on day 14 post-sorting were stained for β-galactosidase (β-gal) expression using Senescence β-galactosidase staining kit (98605, Cell Signaling, United States). EC lysates were used to assess the protein expression of p16, p21, and p53 using Western Blot. The protein expression level was normalized by GAPDH and expressed as percentage of GAPDH. The detailed information on p16, p21, and p53 antibodies was provided in Supplementary Table 1.

### Mitochondrial Function of ECs

To determine EC mitochondrial membrane potential, after overnight cultured with EGM supplemented with DAPI, ECs on day-7 post-sorting were cultured with 1:1 ratio of fresh EGM and JC-1 dye solution (Mitochondria staining kit, Sigma-Aldrich, United States) for 30 min in an incubator at 37°C. Then, cells were washed with DPBS and cultured in fresh EGM. Images of red fluorescence were randomly taken at 50 ms at 20× magnification using Olympus IX73 microscope and Cell Sens Standard software (Olympus, Japan). The fluorescence intensity of each cell was calculated as the overall fluorescence intensity divided by EC number in each image using Image J. Each cell line had three biological replicates and each replicate had 6–8 images analyzed.

To determine mitochondrial structural protein content, EC lysates were used to assess protein expression of mitofusin-1 (Mfn-1), mitofusin-2 (Mfn-2), fission-1 (Fis-1), and ATP5 using Western Blot. The protein expression level was normalized by GAPDH and expressed as percentage of GAPDH. The detailed information on Mfn-1, Mfn-2, and Fis-1 antibodies was provided in Supplementary Table 1.

To determine ATP synthesis efficiency, ECs were cultured in white 96-well plate. On the day of assay, cell culture medium was removed and 100 μl of ATPlite 1 step Luminescence Assay buffer (6016731, PerkinElmer, United States) was added into each well. The intensity of luminescence was determined using Infinite M200 (TECAN, Switzerland) and was normalized to cell number.

### Glycine Homeostasis in ECs

#### Intracellular Glycine Content

Methods of extraction and metabolic profiling for amino acid were described (Newgard et al., 2009; Muoio et al., 2012). ECs on day-7 post-sorting were washed DPBS and were scraped off wells using 300 μL of 0.6% formic acid (Sigma-Aldrich, United States). 100 μL mixture was used for protein content measurement. The remaining mixture was mixed with 200 μL acetonitrile (Sigma-Aldrich, United States). The amino acid extracts were derivatized with 3 M Hydrochloric acid in methanol or butanol (Sigma-Aldrich, United States), dried, and reconstituted in methanol for analysis in Liquid chromatography–mass spectrometry.

Methods of amino acid analysis were modified from Newgard et al. (2009). Briefly, amino acids were separated using a C8 column (Rapid Resolution HT, 4.5 × 50 mm, 1.8 μm, Zorbax SB-C8) on an Agilent 1290 Infinity LC system (Agilent Technologies, CA, United States) coupled with quadrupole-ion trap mass spectrometer (QTRAP 5500, AB Sciex, DC, United States). Mobile phase A (10/90 Water/Acetonitrile) and Mobile phase B (90/10 Water/Acetonitrile) both containing 10 mM of Ammonium formate were used for chromatography separation. The liquid chromatography run was performed at a flow rate of 0.6 mL/min with initial gradient of 20% B for 0.5 min, then ramped to 100% B in 2.5 min, maintained for 0.5 min, followed by re-equilibrated the column to the initial run condition (20% B) for 2 min. All compounds were ionized in positive mode using electrospray ionization. The chromatograms were integrated using MultiQuant 3.0 software (AB Sciex, DC, United States). The amino acid concentration was normalized by protein concentration and presented as μM/mg/ml.

#### Protein Quantification of Glycine Transporters (GlyT) and Serine Hydroxymethyltransferase (SHMT)

Endothelial cell lysates were used to assess the protein expression of GlyT1, GlyT2, cytoplasmic SHMT (cSHMT), and mitochondrial SHMT (mSHMT) using Western Blot. The protein expression level was normalized by GAPDH and expressed as percentage of GAPDH. The detailed information on GlyT1, GlyT2, cSHMT, and mSHMT antibodies was provided in Supplementary Table 1.

### Paracrine Factor Profile of ECs

To assess angiogenic factors released from hiPSC-ECs, 2 × 10^5^ hiPSC-ECs/well were seeded in 12-well plate and cultured in 1 ml endothelial basal medium (EBM) (Lonza, Switzerland) supplemented with 100x B27 for 48 h. The supernatant was collected and used to determine the profiles of angiogenic factors released from hiPSC-ECs using Human Angiogenesis Arrays Q3 kit (Raybio Tech., United States) as described (Ye et al., 2014).

### Endothelial Cell Function *in vivo*

#### Murine Hind-Limb Ischemia (HLI) Model and Treatment

The animal experimental protocol and procedures were approved by the Institutional Animal Care and Use Committee of Singapore Health Services Pte. Ltd. The animal model was developed as previously described (Su et al., 2018). Briefly, 8-week-old NOD-SCID mice (InVivos, Singapore) were anesthetized with 1.5–2% isoflurane and the femoral artery of the right limb was ligated with 6-0 polypropylene sutures. Animals were randomly assigned to treatment with 1.2 10^6^ hiPSC-ECs in 0.1 mL EBM (the hiPSC-EC groups, *n* = 6 for each group) or with 0.1 mL EBM (the basal medium group, *n* = 6). The hiPSC-ECs differentiated from the 4 hiPSC lines or basal medium were administered 3 days after HLI induction via 4 intramuscular injections into the center of the ligated area and the surrounding region along the femoral artery.

#### Laser Doppler Imaging

Mice were anesthetized with 1.5–2% isoflurane, their hind limbs were shaved, and laser Doppler imaging was performed using a PeriScan PIM 3 System (Perimed, Sweden) as described (Su et al., 2018). Measurements in the ischemic (right) limb were normalized to measurements in the non-ischemic (left) limb and expressed as a percentage.

#### Immunohistochemistry

To identify transplanted hiPSC-ECs, a primary antibody specifically against human CD31 (hCD31, mouse anti-human CD31-Biotin) was used and visualized by mouse anti-Biotin-VioBright 515 (both from Miltenyi Biotec, Germany). Fluorescence images were taken with an Olympus IX71 fluorescence microscope.

Neovascularization in ischemic limb was determined as described (Su et al., 2018). Cryosections were stained for CD31 expression (rabbit anti-CD31, Abcam, United States), which targets both human and mouse ECs), to evaluate total vessel density, and smooth muscle actin (SMA) expression (Cy3-conjugated mouse anti-SMA antibodies, Sigma-Aldrich), which targets SMCs, to evaluate arteriole density. Vascular structures that were positive for CD31 expression and for both CD31 and SMA expression were counted for all animals in each group.

### Statistics

Data are presented as mean ± standard deviation (SD). Comparisons among groups were analyzed for significance via one-way analysis of variance (ANOVA) with the Tukey correction. Analyses were performed with SPSS software. A value of *p* < 0.05 was considered significant.

## Results

### Characterization of hiPSCs

P1C1iPSCs, DP2-C8iPSCs, and DP3-C6iPSCs displayed typical morphological features of human embryonic stem cells (hESCs): colonies with a tightly packed appearance and a well-defined edge and cells have prominent nucleoli (Supplementary Figure 1A). They all expressed pluripotent cell markers, including Oct3/4 and TRA-1-60 (Supplementary Figures 1B,C). Karyotyping demonstrated that all three hiPSC lines had normal chromosome number and structure (Supplementary Figure 2A). Teratoma formation assay showed that all three hiPSC lines were able to form teratoma in NOD-SCID mice. Hematoxylin and eosin staining showed that mesoderm (cartilage), ectoderm (epidermis), and endoderm (gastrointestinal glands) lineage cells were formed in teratoma (Supplementary Figure 2B).

### hiPSC-ECs Had Typical EC Characteristics, but dia-hiPSC-ECs Were Less Potent for *in vitro* Angiogenesis

**FIGURE 1:**
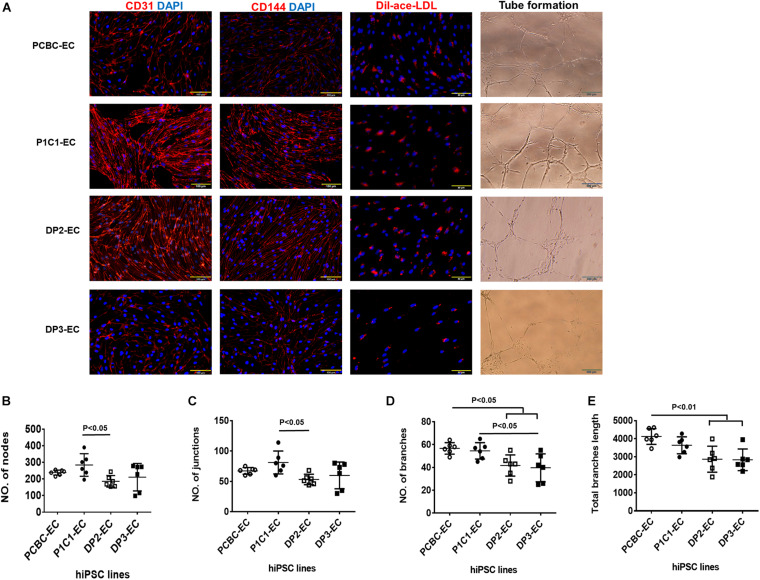
Characterization of hiPSC-ECs. **(A)** ECs differentiated from 4 hiPSC lines were evaluated for the expression of CD31 and CD144, Dil-ace-LDL uptake, and tube formation on Matrigel. Quantifications of numbers of nodes **(B)**, junctions **(C)**, branches **(D),** and total branches length **(E)** formed by hiPSC-ECs on Matrigel (*n* = 6 for each cell line). Values are presented as means ± SD. One-way ANOVA.

Endothelial cells differentiated from 4 hiPSC lines co-expressed CD31 and CD144 (Figure 1A). Functional assessments confirmed that hiPSC-ECs were able to take up DiI-ace-LDL and form tubular structures on Matrigel (Figure 1A). However, the formation of tubular structures was less extensive for DP2-ECs and DP3-ECs than PCBC-ECs and P1C1-ECs. P1C1-ECs formed the highest numbers of nodes (238.33 ± 15.4, *p* < 0.05) and junctions (67.3 ± 5.5, *p* < 0.05) which were significantly higher than DP2-ECs (185.8 ± 34.8 and 53.2 ± 8.6, respectively, Figures 1B,C). PCBC-ECs formed the highest numbers of branches (56.7 ± 5, *p* < 0.05) and had the longest total branch length (4,122 ± 437.2, *p* < 0.01) which were significantly higher than DP2-ECs (41.7 ± 9.3 and 2864.3 ± 719.9, respectively) and DP3-ECs (39.7 ± 12.2 and 2830.8 ± 600, respectively), while P1C1-ECs (54.5 ± 7.2, *p* < 0.05) formed significantly higher number of branches than DP3-ECs only (Figures 1D,E). These results suggest that ECs differentiated from dia-hiPSCs are less potent for *in vitro* angiogenesis.

### Dia-hiPSC-ECs Had a Trend for Reduced NO Production and Increased Endothelin-1 Secretion

**FIGURE 2:**
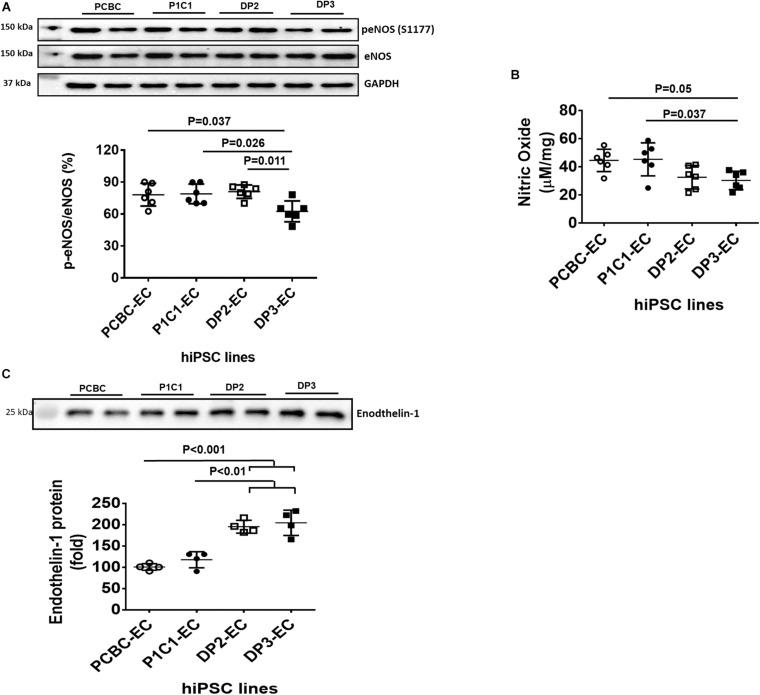
Characterization of nitric oxide (NO) and endothelin-1 produced by hiPSC-ECs. **(A)** Western Blot analysis for quantification of endothelial NO synthase (eNOS) activity (*n* = 6 for each cell line). **(B)** Quantification of NO produced by hiPSC-ECs (*n* = 6 for each cell line). **(C)** Western Blot analysis for quantification of endothelin-1 protein in supernatants of hiPSC-ECs (*n* = 4 for each cell line). Values are presented as means ± SD. One-way ANOVA.

Reduced NO production and increased production of vasoconstrictor, such as endothelin-1, are typical phenotypes of endothelial dysfunction. Thus, we measured and compared NO and endothelin-1 production among 4 hiPSC-EC lines. Western Blot showed that activities of eNOS in PCBC-EC (78.1 ± 11.7%) and P1C1-EC (79.2 ± 9.3%) were similar to that of DP2-EC (77.1 ± 11.1%) and were significantly higher than that of DP3-EC (59.6 ± 11.4%, *p* < 0.05 for both; Figure 2A). Quantification of NO measured by ELISA showed that although PCBC-EC (44.6 ± 7.9 μM/mg) and P1C1-EC (45.4 ± 11.7 μM/mg) had high and DP2-EC (32.6 ± 8.3 μM/mg) and DP3-EC (30.4 ± 6.5 μM/m) had low NO productions, the significant difference was only seen between P1C1-ECs and DP3-ECs (*p* < 0.05; Figure 2B).

Endothelin-1 in supernatant was analyzed using Western Blot (Figure 2C). Endothelin-1 protein in supernatant is significantly increased in DP2-EC (increased by 90 and 66%, respectively) and DP3-EC (increased by 104 and 74%, respectively) compared to PCBC-ECs and P1C1-ECs. This indicates that dia-hiPSC-ECs secrete higher levels of endothelin-1 compared to healthy hiPSC-ECs.

### Disrupted Glycine Homeostasis in Dia-hiPSC-ECs

**FIGURE 3:**
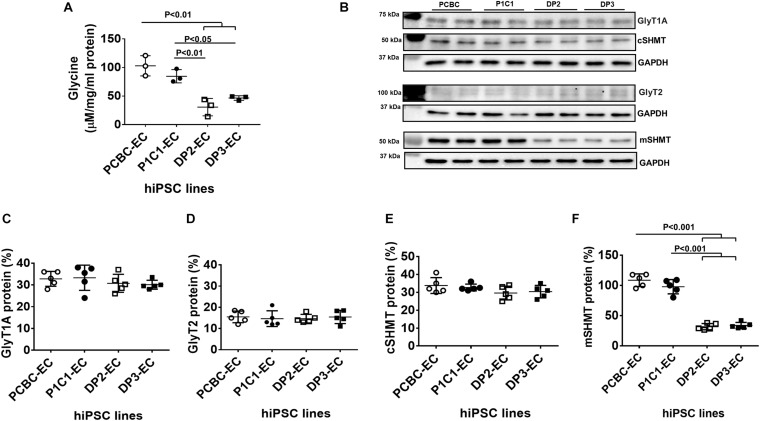
Disrupted glycine homeostasis in dia-hiPSC-ECs. **(A)** Intracellular glycine concentration in hiPSC-ECs measured by liquid chromatography–mass spectrometry (*n* = 3 for each cell line). **(B)** Representative image of Western Blot for GlyT1A, GlyT2, cSHMT, and mSHMT protein expression in hiPSC-ECs. Quantification of GlyT1A **(C)**, GlyT2 **(D)**, cSHMT **(E)**, and mSHMT **(F)** protein expression (*n* = 5 for each cell line for **B,C**). Values are presented as means ± SD. One-way ANOVA.

Metabolomic analysis of amino acid profiles showed that intracellular glycine was significantly lower in dia-hiPSC-ECs compared to healthy hiPSC-ECs (Figure 3A). The intracellular glycine concentration in DP2-EC was 29.6 or 36.2% and DP3-EC was 45.2 or 55% of PCBC-ECs or P1C1-ECs. As intracellular glycine content is maintained by uptake from the media by the transporters GlyT1 and GlyT2 and also by conversion from serine by cSHM and mSHMT, we further measured their protein expression levels. Western Blot showed that the major isoform of GlyT1 is GlyT1A (70.5 kDa), not GlyT1C (78 kDa). Protein expression levels of GlyT1A, GlyT2, and cSHMT were similar among four cells lines (Figures 3B–E).

On the contrary, the protein expression levels of mSHMT were significantly lower in the dia-hiPSC-ECs (DP2-ECs = 32.7 ± 4.4% and 39.7 ± 10.2%) than the healthy hiPSC-ECs (P1C1-EC = 107.1 ± 9.1% and P1C1-EC = 101.9 ± 20.1%, *p* < 0.001 for both), indicating the low intracellular glycine level may be due to low protein expression of mSHMT (Figures 3B,F).

### Dia-hiPSC-ECs Had Slow Growth Rates and Increased Senescence

**FIGURE 4:**
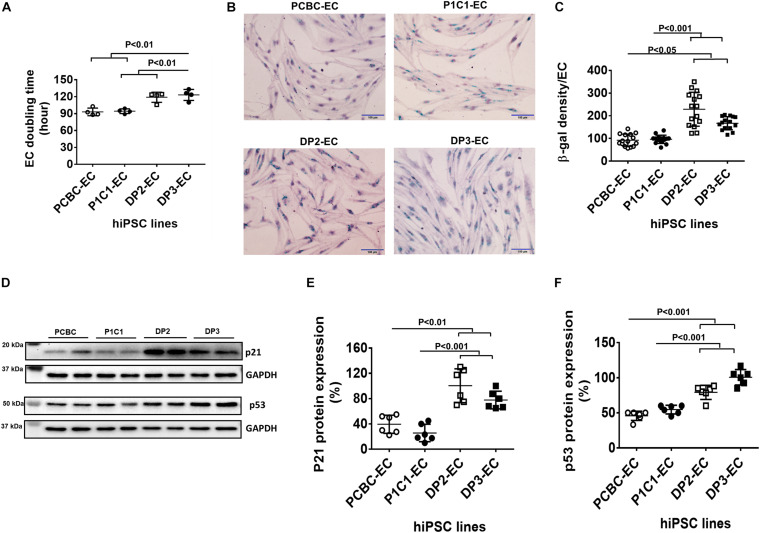
Increased senescence in dia-hiPSC-ECs **(A)**. hiPSC-EC population doubling time (*n* = 4 for each cell line). **(B)** β-gal staining (green color) in hiPSC-ECs which was count-stained with hematoxylin to show cell nuclei. **(C)** Quantification of β-gal density in hiPSC-ECs (*n* = 15 or 16 for each cell line). **(D)** Representative images of Western Blot for p21 and p53 protein expressions. Quantification of p21 **(E)** and p53 **(F)** protein expression (*n* = 6 for each cell line). Values are presented as means ± SD. One-way ANOVA.

Cell doubling time increased by 26 and 30% in DP2-ECs and DP3-ECs, respectively, compared to PCBC-ECs and P1C1-ECs, indicating slower cell growth rate in dia-hiPSC-ECs than healthy hiPSC-ECs (Figure 4A). A cell senescence assay showed that β-gal protein expression significantly increased by 145 and 137%, respectively, in DP2-EC and by 88.9 and 82.7%, respectively, in DP3-EC compared to PCBC-EC and P1C1-EC (Figures 4B,C).

Western Blot showed that p21, not p16 (data not shown), protein expression significantly increased in DP2-ECs (100.42 ± 26.8%) and DP3-ECs (77.8 ± 14%) compared to PCBC-ECs (39.5 ± 14.4%, *p* < 0.01 for both) and P1C1-ECs (25.7 ± 13.8%, *p* < 0.001 for both; Figures 4D,E). Consistently, p53 protein expression also significantly increased in DP2-ECs (72.9 and 44.8%, respectively) and DP3-ECs (117 and 81.2%, respectively) compared to PCBC-ECs and P1C1-ECsy (Figures 4D,F). These results suggest that dia-hiPSC-ECs have increased senescence which may be an underlying cause of slow cell growth rate of dia-hiPSC-ECs due to the accumulation of senescent ECs.

### The Senescence-Associated Secretory Phenotype of Dia-hiPSC-ECs

**FIGURE 5:**
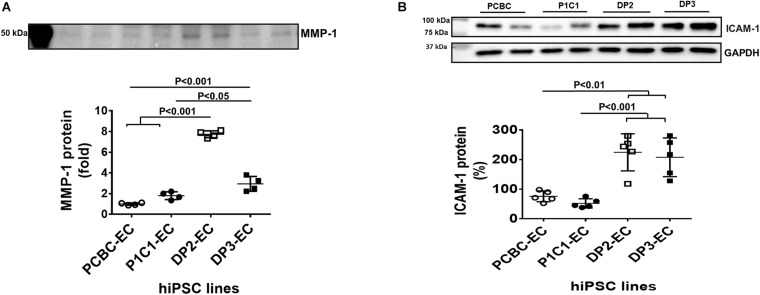
Matrix metalloproteinase-1 (MMP-1) and adhesion molecule expression in hiPSC-ECs. **(A)** Matrix metalloproteinase-1 (MMP-1) content in the supernatant of hiPSC-ECs (*n* = 4 for each cell line). **(B)** Intercellular adhesion molecule-1 (ICAM-1) protein expression in hiPSC-ECs (*n* = 5 for each cell line). Values are presented as means ± SD. One-way ANOVA.

We examined markers that make up the senescence associated secretory phenotype (SASP) (Zeng and Millis, 1996; Coppe et al., 2010; Freitas-Rodriguez et al., 2017). Using the Human Angiogenesis Arrays Q3 Kit, we found that angiopoietin-1 reduced and MMP-1, VEGF, and PECAM-1 increased in the supernatant of dia-hiPSC-ECs as compared to the supernatant of healthy hiPSC-ECs (Supplementary Table 2). Up-regulated MMP-1 and VEGF have been seen in senescent cells and belong to the senescence-associated secretory phenotype (Coppe et al., 2010). Furthermore, Western Blot showed that protein concentration of MMP-1 was significantly higher in the supernatant of the dia-hiPSC-ECs than the healthy hiPSC-ECs (*p* < 0.001: PCBC-EC vs. both DP2-EC and DP3-EC; *p* < 0.001: P1C1-EC vs. DP2-EC; *p* < 0.05: P1C1-EC vs. DP3-EC; Figure 5A). The MMP-1 concentration was about 0.8-folds in P1C1-EC supernatant, 6.74-folds in DP2-EC supernatant, or 1.94-folds in DP3-EC supernatant as compared to PCBC-EC supernatant which was considered as 100%.

#### Increased ICAM-1 Protein Expression

Adhesion molecules, including ICAM-1 and VCAM-1 were determined by Western Blot. Protein expression of ICAM-1 was significantly higher in DP2-EC (224.6 ± 62.6%) and DP3-EC (207.8 ± 65.3%) than in PCBC-EC (75.8 ± 18.2%, *p* < 0.01 for both) and P1C1-EC (52.1 ± 15.65%, *p* < 0.001 for both; Figure 5B). However, VCAM-1 was undetectable (data not shown).

### Mitochondrial Dysfunction in Dia-hiPSC-ECs

**FIGURE 6:**
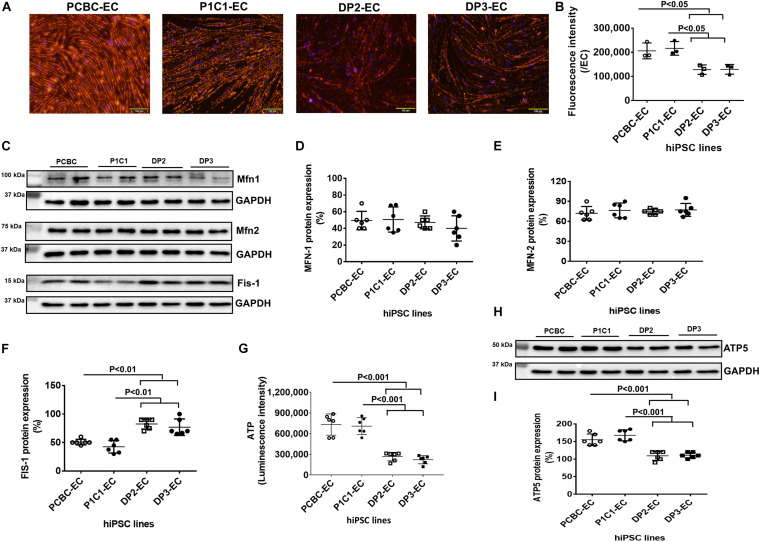
Impaired mitochondrial function in dia-hiPSC-ECs. **(A)** JC-1 staining to visualize mitochondrial membrane potential. **(B)** Quantification of hiPSC-EC mitochondrial membrane potential. **(C)** Representative images of Western Blot for mitochondrial proteins in hiPSC-ECs, including mitofusin-1 (Mfn-1), mitofusin-2 (mfn-2), and Fission-1 (Fis-1). Quantification of MFN-1 **(D)**, MFN-2 **(E)**, and FIS-1 **(F)** protein expression. **(G)** Quantification of ATP production in hiPSC-ECs. **(H)** Western Blot analysis for ATP synthase 5 (ATP5) expression. **(I)** Quantification of ATP5 protein expression (*n* = 6 for each cell line). Values are presented as means ± SD. One-way ANOVA.

JC-1 dye staining of hiPSC-ECs showed that mitochondria of dia-hPSC-ECs had significantly low membrane potential in dia-hiPSC-ECs compared to PCBC-ECs and P1C1-ECs (Figure 6A). The fluorescence intensity reduced significantly by 37.8 and 40.8%, respectively, in DP2-ECs and by 37 and 40.1%, respectively, in DP3-ECs compared to PCBC-ECs (*p* < 0.05 for both) and P1C1-ECs (*p* < 0.05 for both; Figure 6B).

Furthermore, Western Blot showed that although mitochondrial protein expression of Mfn-1 and Mfn-2 were similar among 4 hiPSC-EC lines, protein expression of Fis-1, a protein responsible for mitochondrial fission, was significantly higher in DP2-ECs (82.5 ± 8.8%) and DP3-ECs (76.9 ± 14.55%) compared to PCBC-ECs (50.8 ± 4.9%, *p* < 0.01 for both) and P1C1-ECs (42.5 ± 11%, *p* < 0.01 for both; Figures 6C–F).

ATP production significantly decreased in DP2-EC and DP3-EC as compared with PCBC-EC and P1C1-EC. ATP production indicated by luminescence intensity in DP2-EC was 34 or 35.1% and DP3-EC was 30.3 or 31.3% of PCBC-ECs (*p* < 0.001 for both) or P1C1-ECs (*p* < 0.001 for both; Figure 6G). Consistently, protein expression of ATP5, a subunit of ATP synthase, significantly decreased in dia-hiPSC-ECs (DP2-EC = 109.2 ± 12.8% and DP3-EC = 110.8 ± 8%) compared to healthy hiPSC-ECs (PCBC-EC = 155 ± 16.4%, *p* < 0.001 for both and P1C1 = 167.5 ± 15.7%, *p* < 0.001 for both; Figures 6H,I). These results suggest that dia-hiPSC-ECs have impaired mitochondrial function as evidenced by low mitochondrial membrane potential and ATP production, decreased ATP5 protein expression, and increased Fis-1 protein expression.

### dia-hiPSC-EC Have Poor Angiogenic Potential for Treatment of HLI

**FIGURE 7:**
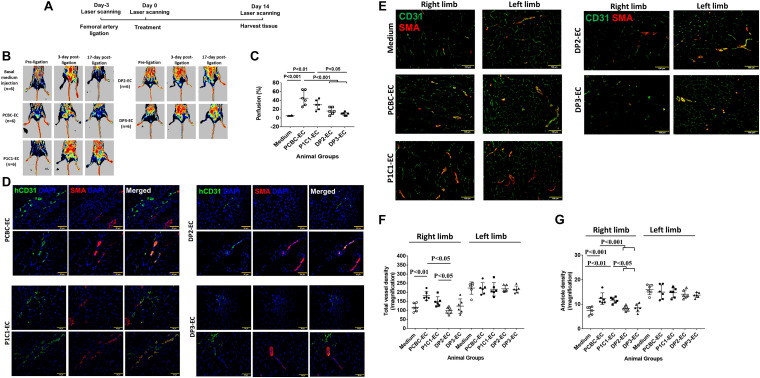
Assessment of hiPSC-EC function in a mouse model of hind-limb ischemia (HLI) *in vivo*. **(A)** A schematic diagram of the HLI model and treatment. **(B)** Laser Doppler imaging of mouse limbs before femoral artery ligation, 3 days (i.e., at the time of treatment administration), and 17 days (i.e., at the 14 days medium or cell injection) after femoral artery ligation. **(C)** Recovery of right limb perfusion was expressed as a percentage of measurements in the uninjured contralateral limb (*n* = 6 for each cell line). **(D)** Fluorescence staining for human-specific CD31 (hCD31) and smooth muscle actin (SMA) expression in the injured limbs of hiPSC-EC–treated animals (Bar = 50 μm). **(E)** Fluorescence staining for CD31 and smooth muscle actin (SMA) in the ischemic limb (right leg) and uninjured limb (left leg) of animals treated with basal medium or hiPSC-ECs after femoral artery ligation (Bar = 100 μm). **(F)** Vessel density and **(G)** arteriole density in ischemic limbs (right limb) and uninjured contralateral limbs (left limb) (*n* = 6 for each cell line). Values are presented as means ± SD. One-way ANOVA.

The potential of dia-hiPSC-ECs for treatment of ischemic disease was evaluated in a mouse model of HLI (Figure 7A; Su et al., 2018). Perfusion was barely detectable in mouse limbs of the basal medium group at 14 days post-treatment. However, perfusion was recovered to 45 or 35% in the ischemic limbs of the PCBC-EC or P1C1-EC group, which was not only significantly greater than the extent of recovery in the basal medium injected mice (5.7 ± 1%, *p* < 0.001 or *p* < 0.01), but also than that in the DP2-EC (15.8 ± 8%, *p* < 0.001 vs. PCBC-EC group) or DP3-EC group (12.7 ± 3.8%, *p* < 0.001 vs. PCBC-EC group or *p* < 0.05 vs. P1C1-EC group; Figures 7B,C).

Tissue sections stained for the specific hCD31 and SMA indicated that the transplanted hiPSC-ECs were present both in capillaries and smooth muscle cell coated arterioles in all animal groups that received hiPSC-EC transplantation (Figure 7D), suggesting that transplanted hiPSC-ECs can contribute to capillary and arteriole formation in ischemic limbs.

Assessments in sections stained for CD31 (detecting both hiPSC-EC and mouse EC) and SMA (Figure 7E) indicated that total vessel density was significantly higher in the ischemic limbs of animals in the PCBC-EC group (180.5 ± 24.7) than in the basal medium (115.5 ± 22.9, *p* < 0.01) or DP2-EC (98.9 ± 18.5, *p* < 0.05) group, while total vessel density of the P1C1 group (was only significantly higher than the DP2-EC group (Figure 7F). Arteriole densities in the ischemic limbs of animals were significantly higher in the PCBC-EC (12.3 ± 2.3) or P1C1 (11.6 ± 1.2) groups than the basal medium (7.4 ± 1.6, *p* < 0.001 vs. PCBC-EC group or *p* < 0.01 vs. P1C1-EC group), DP2-EC (8.2 ± 1, *p* < 0.001 or *p* < 0.05), and DP3-EC (8.5 ± 1.6, *p* < 0.001 or *p* < 0.05) groups (Figure 7G). The increased arteriole density may be due to the angiopoietin-1 secreted by healthy hiPSC-ECs after transplantation (Supplementary Table 2). Collectively, these observations suggest that transplanted dia-hiPSC-ECs had poor angiogenic potential in restoring perfusion and stimulating neovascularization in ischemic limb.

## Discussion

We characterized hiPSC-ECs derived from patients with T2DM and identified feature phenotypes in two dia-hiPSC-EC lines: disrupted glycine homeostasis, increased senescence, and impaired mitochondrial function and angiogenic potential.

Diabetes causes endothelial dysfunction (Avogaro et al., 2011; Giraldo-Grueso and Echeverri, 2020). Some of the mediators of endothelial dysfunction include reduced bioavailable NO, increased production of vasoconstrictors, such as endothelin-1 and angiotensin II, and dysregulated inflammation or thrombosis (DeFronzo et al., 2015; Palmer A.K. et al., 2015; Yan-Do and MacDonald, 2017; Alves et al., 2019; Kruger-Genge et al., 2019; Rashvand et al., 2019; Giraldo-Grueso and Echeverri, 2020). Endothelial dysfunction is considered as an early step in the cascade of adverse events that lead to atherosclerosis and ischemic heart disease (Giraldo-Grueso and Echeverri, 2020). In addition, diabetes causes impaired angiogenesis (Kota et al., 2012; Fadini et al., 2019) and induces endothelial senescence (Shosha et al., 2018; Bitar, 2019) and mitochondrial dysfunction (Teodoro et al., 2018; Yaribeygi et al., 2019), all of which have been associated with diabetic complications and are being investigated as therapeutic targets.

hiPSCs generated human ECs not only can be used for treatment of ischemic diseases, but also can be used for modeling diseases and drug screening and discovering *in vitro* (Liu et al., 2018; Rowe and Daley, 2019; Williams and Wu, 2019). Although ECs differentiated from hiPSCs of pulmonary hypertension and Hutchinson–Gilford progeria syndrome have been studied (Zhang et al., 2011; Gu et al., 2017; Sa et al., 2017; Matrone et al., 2019), few studies have characterized the function of iPSC derived ECs in diabetes or metabolic syndrome in rodents (Takenaka-Ninagawa et al., 2014; Gu et al., 2015) and human (Vila Gonzalez et al., 2020). The endothelial dysfunction of mouse iPSC derived from obesity is due to decreased NO production (Gu et al., 2015). Only one study has investigated ECs derived from hiPSCs of patients with diabetic macular edema (Vila Gonzalez et al., 2020). The results of this work were able to recapitulate clinical evaluations of anti-VEGF responsiveness *in vitro*. Thus, feature phenotypes are largely unknown in hiPSC-ECs from patients with diabetes. In this study, we characterized dia-hiPSC-ECs and found that they not only recapitulated several phenotypes of endothelial dysfunction, such as increased vasoconstrictor (endothelin-1) and adhesion molecule (ICAM-1) production and impaired angiogenesis, but also showed disrupted glycine homeostasis, increased senescence, and impaired mitochondrial function.

Although decreased NO production is a feature phenotype of endothelial dysfunction, significantly decreased eNOS activity and NO production were only found in DP3-ECs in this study. However, endothelin-1 and ICAM-1 production significantly increased in both dia-hiPSC-EC lines. These results indicate that increased vasoconstrictor and adhesion molecule expression may be more common phenotypes than reduced NO production in dia-hiPSC-ECs.

One signature phenotype we found in this study is the disrupted glycine homeostasis, including low intracellular glycine content. This is consistent with clinical findings of serum glycine level which is lower not only in T2DM patients, but also in patients with impaired glucose tolerance (Wang-Sattler et al., 2012; Drabkova et al., 2015; Palmer N.D. et al., 2015). Serum glycine concentration was negatively associated with steady-state plasma glucose (Seibert et al., 2015) and has been identified as a serum biomarker of both insulin sensitivity and regional fat mass in functionally limited older adults (Lustgarten et al., 2013). Low intracellular glycine level may compromise vascular endothelial growth factor signaling in ECs and lead to poor angiogenic potential and mitochondrial function (Guo et al., 2017). The observed low intracellular glycine content may be due to low protein expression levels of mSHTM. Intracellular glycine homeostasis is modulated by GlyT (GlyT1 and GlyT2) and SHMT (cSHMT and mSHMT). Except for mSHMT, the other three protein expression levels are similar between the healthy hiPSC-ECs and dia-hiPSC-ECs. Future studies are needed to explore the mechanism of how disrupted intracellular glycine homeostasis causes endothelial dysfunction in dia-hiPSC-ECs for a better understanding the role of glycine homeostasis in endothelial function in diabetes.

Increased senescence is found in dia-hiPSC-ECs. Feature characteristics of senescent dia-hiPSC-ECs include up-regulated β-gal, p21, and p53 protein expression and increased secretion of MMP-1 and VEGF which are components of a SASP (Coppe et al., 2010), and impaired mitochondrial function. Mitochondrial dysfunction is composed of up-regulated Fis-1 protein expression, decreased mitochondrial membrane potential, ATP synthase protein expression, and ATP production in dia-hiPSC-ECs. Although senescence may be a causal role in mitochondrial dysfunction (Korolchuk et al., 2017), it may be that mitochondrial dysfunction itself leads to senescence (Wiley et al., 2016). Increased senescence in dia-hiPEC-ECs may be due to the high protein expression levels of p53 and p21 as the p53/p21 pathway leads to the inhibition of Cdk4/6 activity and contributes to senescence (Sharpless and Sherr, 2015). Poor cell viability associated with senescent dia-hiPSC-ECs may result in poor angiogenic potential *in vitro* and *in vivo*.

One limitation of the current study is the number of hiPSC lines used. We only used 2 hiPSC lines each of healthy subjects and patients with T2DM. Although robust difference was observed in glycine homeostasis, endothelial senescence, mitochondrial function, angiogenic potential in dia-hiPSC-ECs as compared with healthy hiPSC-ECs, they may be limited in 2 dia-hiPSC-EC lines used in this study.

In conclusion, we identified feature phenotypes in dia-hiPSC-ECs: disrupted glycine homeostasis, increased senescence, impaired mitochondrial function and angiogenesis. Efforts are needed to find the key causal factors and the molecular mechanisms contributing to the onset and development of disrupted glycine homeostasis and senescence in dia-hiPEC-ECs. The features identified here may serve as targets for new therapeutic strategies for the treatment of endothelial dysfunction in diabetes.

## Data Availability Statement

The data underlying this article are available in the article and in its online Supplementary Material.

## Ethics Statement

The animal study was reviewed and approved by the Institutional Animal Care and Use Committee of Singapore Health Services Pte. Ltd.

## Author Contributions

LS, XK, SL, and YG: collection and/or assembly of data and data analysis and interpretation. J-PK: manuscript writing and editing. XS and JM: provision of study material and manuscript writing. LY: conceptualization, performing experiments, data collection, writing, and financial support. All authors contributed to the article and approved the submitted version.

## Conflict of Interest

The authors declare that the research was conducted in the absence of any commercial or financial relationships that could be construed as a potential conflict of interest.
