# Supplementary material for: Diabetic Endothelial Cells Differentiated From Patient iPSCs Show Dysregulated Glycine Homeostasis and Senescence Associated Phenotypes
Source: Front Cell Dev Biol. 2021 May 31;9:667252. doi: 10.3389/fcell.2021.667252 (PMC8201091; doi:10.3389/fcell.2021.667252)
Supplement: Supplementary file 1 [file Presentation_1.PPT]

## Slide 1
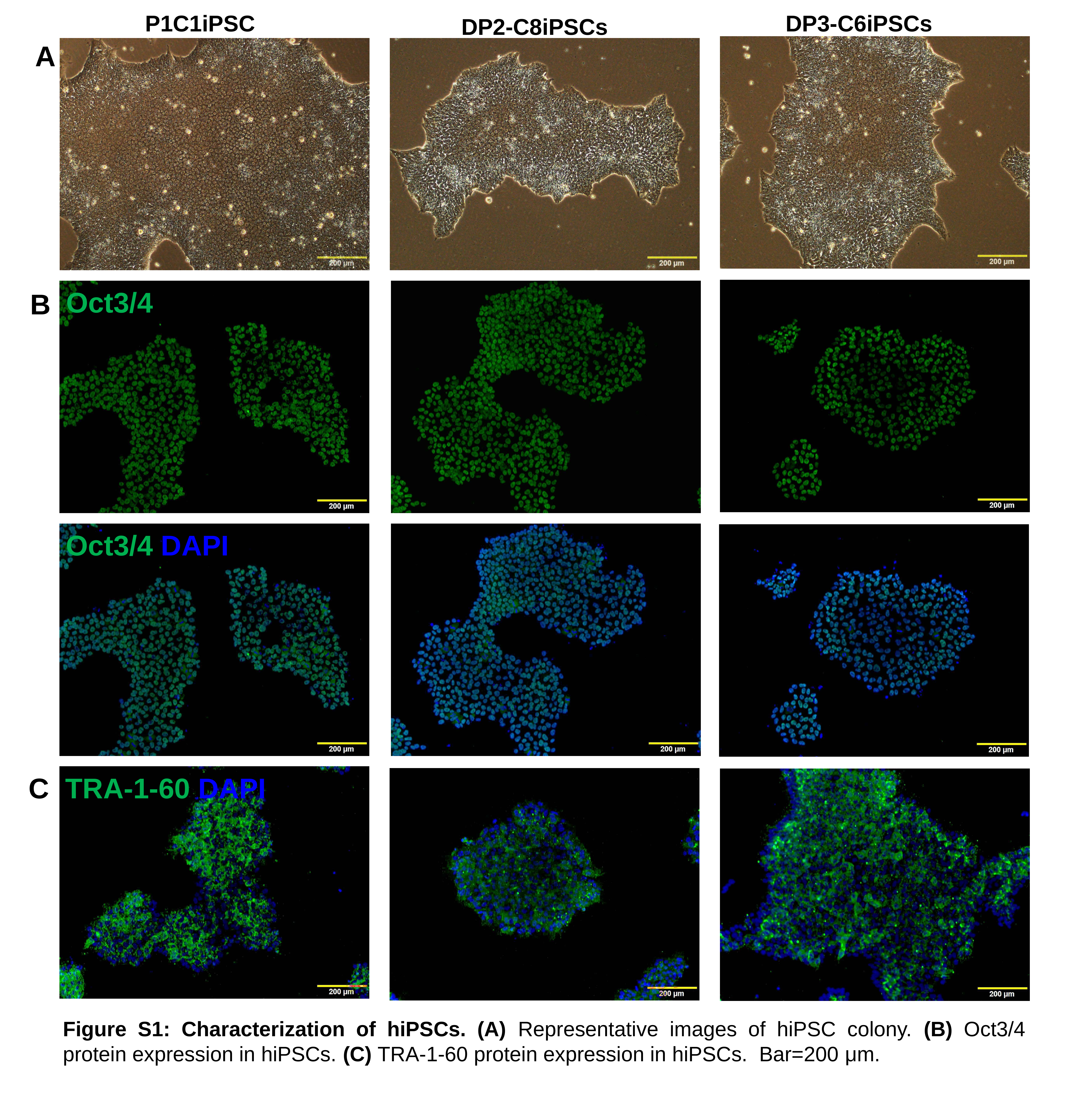

DP3-C6iPSCs
P1C1iPSC
DP2-C8iPSCs
A
Oct3/4
B
Oct3/4 DAPI
C
TRA-1-60 DAPI
Figure S1: Characterization of hiPSCs. (A) Representative images of hiPSC colony. (B) Oct3/4 protein expression in hiPSCs. (C) TRA-1-60 protein expression in hiPSCs. Bar=200 μm.

## Slide 2
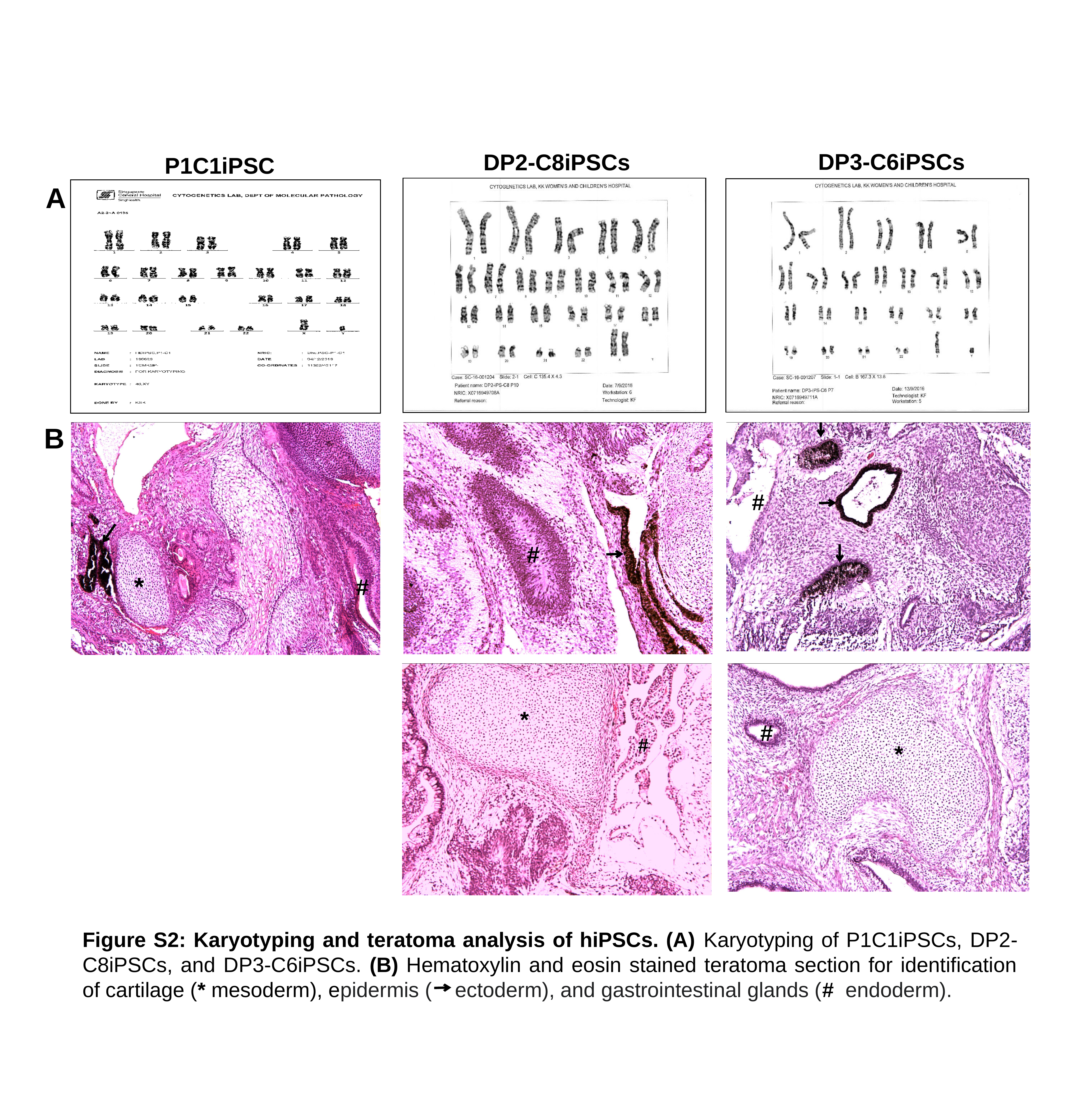

DP3-C6iPSCs
DP2-C8iPSCs
P1C1iPSC
A
B
#
#
*
#
*
#
#
*
Figure S2: Karyotyping and teratoma analysis of hiPSCs. (A) Karyotyping of P1C1iPSCs, DP2-C8iPSCs, and DP3-C6iPSCs. (B) Hematoxylin and eosin stained teratoma section for identification of cartilage (* mesoderm), epidermis ( ectoderm), and gastrointestinal glands (# endoderm).

## Slide 3
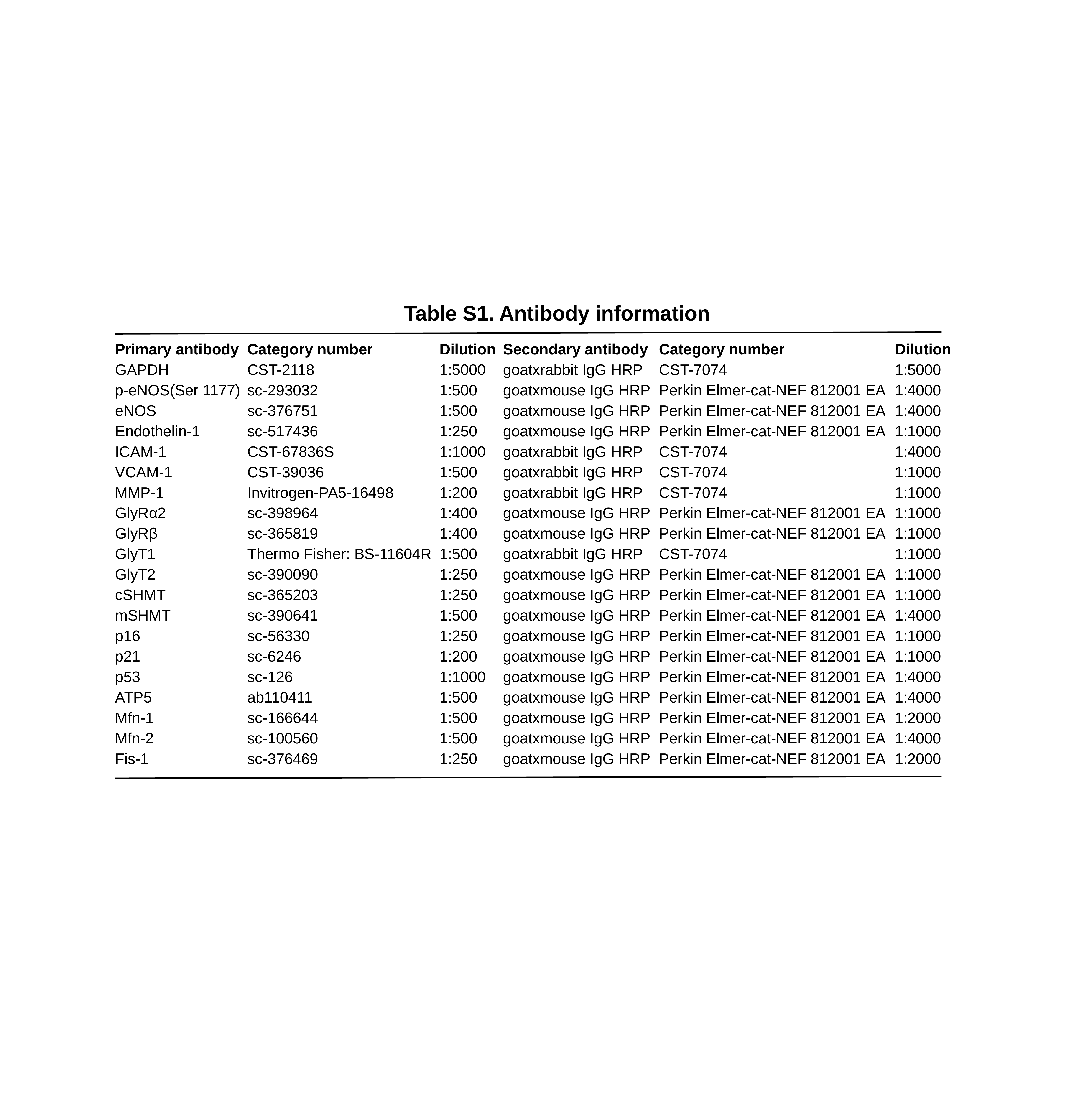

Table S1. Antibody information
| Primary antibody | Category number | Dilution | Secondary antibody | Category number | Dilution |
| --- | --- | --- | --- | --- | --- |
| GAPDH | CST-2118 | 1:5000 | goatxrabbit IgG HRP | CST-7074 | 1:5000 |
| p-eNOS(Ser 1177) | sc-293032 | 1:500 | goatxmouse IgG HRP | Perkin Elmer-cat-NEF 812001 EA | 1:4000 |
| eNOS | sc-376751 | 1:500 | goatxmouse IgG HRP | Perkin Elmer-cat-NEF 812001 EA | 1:4000 |
| Endothelin-1 | sc-517436 | 1:250 | goatxmouse IgG HRP | Perkin Elmer-cat-NEF 812001 EA | 1:1000 |
| ICAM-1 | CST-67836S | 1:1000 | goatxrabbit IgG HRP | CST-7074 | 1:4000 |
| VCAM-1 | CST-39036 | 1:500 | goatxrabbit IgG HRP | CST-7074 | 1:1000 |
| MMP-1 | Invitrogen-PA5-16498 | 1:200 | goatxrabbit IgG HRP | CST-7074 | 1:1000 |
| GlyRα2 | sc-398964 | 1:400 | goatxmouse IgG HRP | Perkin Elmer-cat-NEF 812001 EA | 1:1000 |
| GlyRβ | sc-365819 | 1:400 | goatxmouse IgG HRP | Perkin Elmer-cat-NEF 812001 EA | 1:1000 |
| GlyT1 | Thermo Fisher: BS-11604R | 1:500 | goatxrabbit IgG HRP | CST-7074 | 1:1000 |
| GlyT2 | sc-390090 | 1:250 | goatxmouse IgG HRP | Perkin Elmer-cat-NEF 812001 EA | 1:1000 |
| cSHMT | sc-365203 | 1:250 | goatxmouse IgG HRP | Perkin Elmer-cat-NEF 812001 EA | 1:1000 |
| mSHMT | sc-390641 | 1:500 | goatxmouse IgG HRP | Perkin Elmer-cat-NEF 812001 EA | 1:4000 |
| p16 | sc-56330 | 1:250 | goatxmouse IgG HRP | Perkin Elmer-cat-NEF 812001 EA | 1:1000 |
| p21 | sc-6246 | 1:200 | goatxmouse IgG HRP | Perkin Elmer-cat-NEF 812001 EA | 1:1000 |
| p53 | sc-126 | 1:1000 | goatxmouse IgG HRP | Perkin Elmer-cat-NEF 812001 EA | 1:4000 |
| ATP5 | ab110411 | 1:500 | goatxmouse IgG HRP | Perkin Elmer-cat-NEF 812001 EA | 1:4000 |
| Mfn-1 | sc-166644 | 1:500 | goatxmouse IgG HRP | Perkin Elmer-cat-NEF 812001 EA | 1:2000 |
| Mfn-2 | sc-100560 | 1:500 | goatxmouse IgG HRP | Perkin Elmer-cat-NEF 812001 EA | 1:4000 |
| Fis-1 | sc-376469 | 1:250 | goatxmouse IgG HRP | Perkin Elmer-cat-NEF 812001 EA | 1:2000 |

## Slide 4
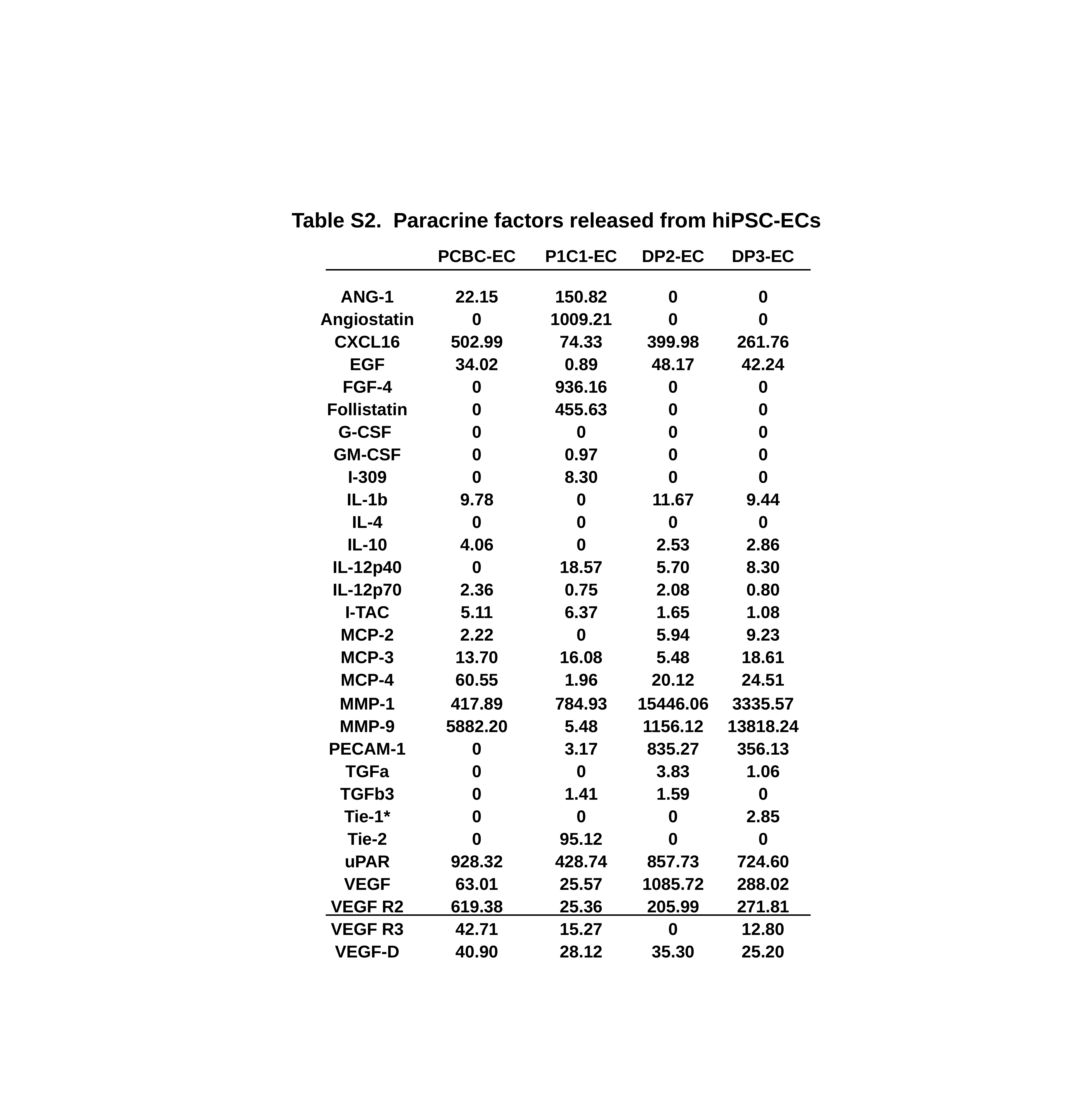

Table S2. Paracrine factors released from hiPSC-ECs
| | PCBC-EC | P1C1-EC | DP2-EC | DP3-EC |
| --- | --- | --- | --- | --- |
| ANG-1 | 22.15 | 150.82 | 0 | 0 |
| Angiostatin | 0 | 1009.21 | 0 | 0 |
| CXCL16 | 502.99 | 74.33 | 399.98 | 261.76 |
| EGF | 34.02 | 0.89 | 48.17 | 42.24 |
| FGF-4 | 0 | 936.16 | 0 | 0 |
| Follistatin | 0 | 455.63 | 0 | 0 |
| G-CSF | 0 | 0 | 0 | 0 |
| GM-CSF | 0 | 0.97 | 0 | 0 |
| I-309 | 0 | 8.30 | 0 | 0 |
| IL-1b | 9.78 | 0 | 11.67 | 9.44 |
| IL-4 | 0 | 0 | 0 | 0 |
| IL-10 | 4.06 | 0 | 2.53 | 2.86 |
| IL-12p40 | 0 | 18.57 | 5.70 | 8.30 |
| IL-12p70 | 2.36 | 0.75 | 2.08 | 0.80 |
| I-TAC | 5.11 | 6.37 | 1.65 | 1.08 |
| MCP-2 | 2.22 | 0 | 5.94 | 9.23 |
| MCP-3 | 13.70 | 16.08 | 5.48 | 18.61 |
| MCP-4 | 60.55 | 1.96 | 20.12 | 24.51 |
| MMP-1 | 417.89 | 784.93 | 15446.06 | 3335.57 |
| MMP-9 | 5882.20 | 5.48 | 1156.12 | 13818.24 |
| PECAM-1 | 0 | 3.17 | 835.27 | 356.13 |
| TGFa | 0 | 0 | 3.83 | 1.06 |
| TGFb3 | 0 | 1.41 | 1.59 | 0 |
| Tie-1\* | 0 | 0 | 0 | 2.85 |
| Tie-2 | 0 | 95.12 | 0 | 0 |
| uPAR | 928.32 | 428.74 | 857.73 | 724.60 |
| VEGF | 63.01 | 25.57 | 1085.72 | 288.02 |
| VEGF R2 | 619.38 | 25.36 | 205.99 | 271.81 |
| VEGF R3 | 42.71 | 15.27 | 0 | 12.80 |
| VEGF-D | 40.90 | 28.12 | 35.30 | 25.20 |
